# 3,3′-(Ethane-1,2-di­yl)bis­(6-methyl-3,4-dihydro-2*H*-1,3-benzoxazine)

**DOI:** 10.1107/S1600536811027139

**Published:** 2011-07-13

**Authors:** Augusto Rivera, Jairo Camacho, Jaime Ríos-Motta, Michaela Pojarová, Michal Dušek

**Affiliations:** a Departamento de Química, Universidad Nacional de Colombia, Ciudad, Universitaria, Bogotá, Colombia; b Institute of Physics, v.v.i, AS CR, Na Slovance 2, 182 21 Prague 8, Czech Republic

## Abstract

The asymmetric unit of the title compound, C_20_H_24_N_2_O_2_, contains one half-mol­ecule, which is completed by inversion symmetry. In the crystal, mol­ecular chains are formed through non-classical C—H⋯O hydrogen bonds, formed between axial H atoms of the oxazine ring and a O atom of a neighboring mol­ecule.

## Related literature

For the synthesis, see: Rivera *et al.* (1994 ▶). For a related structure, see: Rivera *et al.* (2010 ▶). For uses of benzoxazines in polymer science, see Yaggi *et al.* (2009 ▶). For the biological activity of bis-benzoxazine compounds, see: Billmann & Dorman (1963 ▶); Heinisch *et al.* (2002 ▶).
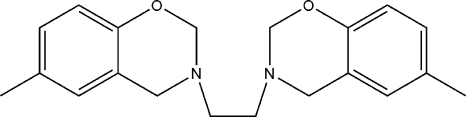


## Experimental

### 

#### Crystal data

C_20_H_24_N_2_O_2_
*M* *_r_* = 324.41
Monoclinic, 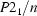

*a* = 8.5042 (1) Å
*b* = 5.8558 (1) Å
*c* = 16.5519 (2) Å
β = 95.899 (1)°
*V* = 819.90 (2) Å^3^
*Z* = 2
Cu *K*α radiation
μ = 0.68 mm^−1^
*T* = 130 K
0.50 × 0.33 × 0.20 mm

#### Data collection

Xcalibur, Atlas, Gemini ultra diffractometer
Absorption correction: analytical (*CrysAlis PRO*; Agilent, 2011 ▶) *T*_min_ = 0.384, *T*_max_ = 0.668
7156 measured reflections
1452 independent reflections
1429 reflections with *I* > 2σ(*I*)
*R*_int_ = 0.013

#### Refinement

*R*[*F* ^2^ > 2σ(*F* ^2^)] = 0.034
*wR*(*F* ^2^) = 0.087
*S* = 1.03
1452 reflections
111 parameters
H-atom parameters constrained
Δρ_max_ = 0.21 e Å^−3^
Δρ_min_ = −0.16 e Å^−3^

### 

Data collection: *CrysAlis PRO* (Agilent, 2011 ▶); cell refinement: *CrysAlis PRO*; data reduction: *CrysAlis PRO*; program(s) used to solve structure: *SHELXS97* (Sheldrick, 2008 ▶); program(s) used to refine structure: *SHELXL97* (Sheldrick, 2008 ▶); molecular graphics: *DIAMOND* (Brandenburg & Putz, 2005 ▶); software used to prepare material for publication: *publCIF* (Westrip, 2010 ▶).

## Supplementary Material

Crystal structure: contains datablock(s) I, global. DOI: 10.1107/S1600536811027139/qm2015sup1.cif

Structure factors: contains datablock(s) I. DOI: 10.1107/S1600536811027139/qm2015Isup2.hkl

Supplementary material file. DOI: 10.1107/S1600536811027139/qm2015Isup3.cml

Additional supplementary materials:  crystallographic information; 3D view; checkCIF report

## Figures and Tables

**Table 1:** Hydrogen-bond geometry (Å, °)

*D*—H⋯*A*	*D*—H	H⋯*A*	*D*⋯*A*	*D*—H⋯*A*
C2—H2*A*⋯O1^i^	0.97	2.57	3.425 (1)	147

Symmetry code: (i) 
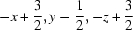
.

**Fig. 1.**
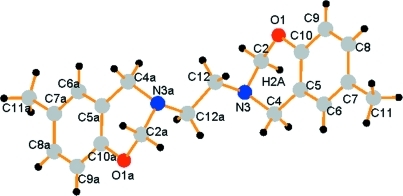
Molecule of the title compound with atom-labeling scheme.

**Fig. 2.**
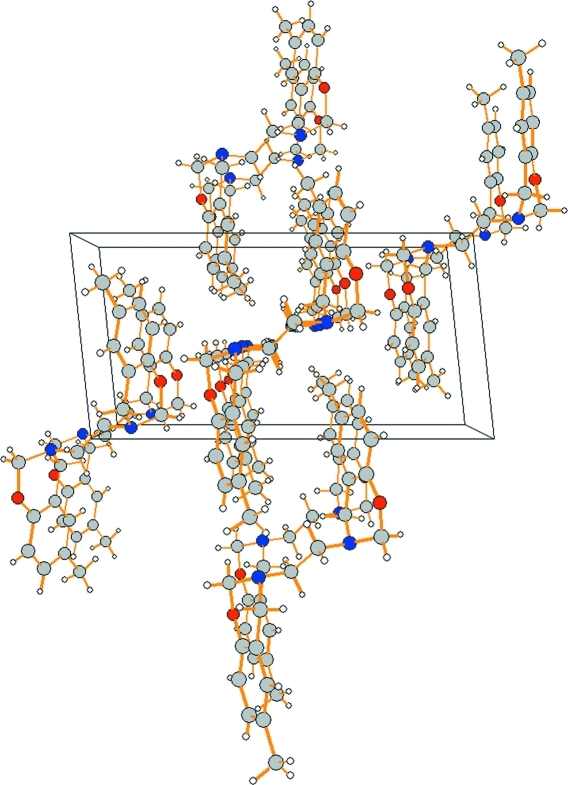
Packing of the molecules of the title compound view along b.

## supplementary crystallographic information

### Comment

In the title compound, C_20_H_24_N_2_O_2_, the asymmetric unit contains one-half of the molecule, which is related to the other half by a centre of inversion located at the mid-point of the central C12—C12*a* bond (see Fig.1). The unit cell contains two molecules. Unlike the related structure Rivera *et al.* (2010), which crystallized in space group C2/c the title compound crystallizes in the P2_1._/n space group.

The molecule contains two 1,3-benzoxazine units linked by a CH_2_CH_2_ spacer at the 3 position of heterocyclic ring. The bond lengths and angles are within normal ranges, whereas the observed C—O bond length [1.376 (1) Å and 1.453 (1) Å] are considerably shortened in relation to related structure (Rivera *et al.*, 2010) [1.421 (2) Å and 1.529 (2) Å]. The C—N bond length [1.429 (1) Å] in the N—CH_2_—O segment is more agreement with the typical than the related structure (Rivera *et al.*, 2010) [1.369 (2) Å]. This information indicates minor influence of the anomeric effect in the title compound. The heterocyclic ring adopts a cyclohexene-like half chair conformation. In the crystal structure, molecules are linked by non clasical intermolecular C—H···.O interactions between H2A and O1 of a neighboring molecule. This establishes crystal packing into 1-D extended chains along the *b*-axis (see Fig. 2).

### Experimental

To a stirred solution of 1,3-bis(2'-hydroxy-5'-methyl-benzyl)imidazolidine (1 mmol) in dioxne is added slowly dropwise formaldehyde solution 40% (1 mmol) (8 ml, 0.11 mmol) and the mixture gently warned at 40–42 °C until a precipitate appeared. The product was filtered and washed with alcohol and water. Recrystallization of solid from ethyl acetate gives title compound (yield 82%). *M*.p. 401–402 K.

### Refinement

All hydrogen atoms were discernible in difference Fourier maps and could be refined to reasonable geometry. According to common practice H atoms attached to C atoms were nevertheless kept in ideal positions during the refinement. The isotropic atomic displacement parameters of hydrogen atoms were evaluated as 1.2**U*_eq_ of the parent atom.

### Crystal data

**Table:**

C_20_H_24_N_2_O_2_	*F*(000) = 348
*M**_r_* = 324.41	*D*_x_ = 1.314 Mg m^−^^3^
Monoclinic, *P*2_1_/*n*	Cu *K*α radiation, λ = 1.5418 Å
Hall symbol: -P 2yn	Cell parameters from 6632 reflections
*a* = 8.5042 (1) Å	θ = 5.2–67.0°
*b* = 5.8558 (1) Å	µ = 0.68 mm^−^^1^
*c* = 16.5519 (2) Å	*T* = 130 K
β = 95.899 (1)°	Plate, colourless
*V* = 819.90 (2) Å^3^	0.50 × 0.33 × 0.20 mm
*Z* = 2	

### Data collection

**Table:**

Xcalibur, Atlas, Gemini ultra diffractometer	1452 independent reflections
Radiation source: Enhance Ultra (Cu) X-ray Source	1429 reflections with *I* > 2σ(*I*)
mirror	*R*_int_ = 0.013
Detector resolution: 10.3784 pixels mm^-1^	θ_max_ = 67.1°, θ_min_ = 5.4°
Rotation method data acquisition using ω scans	*h* = −10→10
Absorption correction: analytical (*CrysAlis PRO*; Agilent, 2011)	*k* = −7→6
*T*_min_ = 0.384, *T*_max_ = 0.668	*l* = −18→19
7156 measured reflections	

### Refinement

**Table:**

Refinement on *F*^2^	Secondary atom site location: difference Fourier map
Least-squares matrix: full	Hydrogen site location: inferred from neighbouring sites
*R*[*F*^2^ > 2σ(*F*^2^)] = 0.034	H-atom parameters constrained
*wR*(*F*^2^) = 0.087	*w* = 1/[σ^2^(*F*_o_^2^) + (0.0446*P*)^2^ + 0.3441*P*] where *P* = (*F*_o_^2^ + 2*F*_c_^2^)/3
*S* = 1.03	(Δ/σ)_max_ = 0.001
1452 reflections	Δρ_max_ = 0.21 e Å^−^^3^
111 parameters	Δρ_min_ = −0.16 e Å^−^^3^
0 restraints	Extinction correction: *SHELXL97* (Sheldrick, 2008), Fc^*^=kFc[1+0.001xFc^2^λ^3^/sin(2θ)]^-1/4^
Primary atom site location: structure-invariant direct methods	Extinction coefficient: 0.033 (2)

### Special details

**Table:**

Geometry. All e.s.d.'s (except the e.s.d. in the dihedral angle between two l.s. planes) are estimated using the full covariance matrix. The cell e.s.d.'s are taken into account individually in the estimation of e.s.d.'s in distances, angles and torsion angles; correlations between e.s.d.'s in cell parameters are only used when they are defined by crystal symmetry. An approximate (isotropic) treatment of cell e.s.d.'s is used for estimating e.s.d.'s involving l.s. planes.
Refinement. Refinement of *F*^2^ against ALL reflections. The weighted *R*-factor *wR* and goodness of fit *S* are based on *F*^2^, conventional *R*-factors *R* are based on *F*, with *F* set to zero for negative *F*^2^. The threshold expression of *F*^2^ > σ(*F*^2^) is used only for calculating *R*-factors(gt) *etc*. and is not relevant to the choice of reflections for refinement. *R*-factors based on *F*^2^ are statistically about twice as large as those based on *F*, and *R*- factors based on ALL data will be even larger. The H atoms were all located in a difference map, but those attached to carbon atoms were repositioned geometrically. The isotropic temperature parameters of hydrogen atoms were calculated as 1.2**U*_eq_ of the parent atom.

### Fractional atomic coordinates and isotropic or equivalent isotropic displacement parameters (Å^2^)

**Table:**

	*x*	*y*	*z*	*U*_iso_*/*U*_eq_	
O1	0.72879 (9)	0.13355 (13)	0.81920 (5)	0.0219 (2)	
C2	0.88891 (13)	0.0460 (2)	0.82084 (7)	0.0207 (3)	
H2A	0.8959	−0.0491	0.7734	0.025*	
H2B	0.9608	0.1735	0.8174	0.025*	
N3	0.93877 (10)	−0.08416 (16)	0.89191 (5)	0.0191 (3)	
C4	0.84012 (13)	−0.29037 (19)	0.89165 (6)	0.0196 (3)	
H4A	0.8583	−0.3650	0.9441	0.023*	
H4B	0.8708	−0.3956	0.8508	0.023*	
C5	0.66601 (13)	−0.23570 (19)	0.87416 (6)	0.0189 (3)	
C6	0.54855 (13)	−0.3874 (2)	0.89236 (6)	0.0205 (3)	
H6	0.5781	−0.5261	0.9168	0.025*	
C7	0.38861 (13)	−0.3384 (2)	0.87522 (7)	0.0220 (3)	
C8	0.34747 (13)	−0.1288 (2)	0.83884 (7)	0.0245 (3)	
H8	0.2412	−0.0917	0.8271	0.029*	
C9	0.46154 (14)	0.0245 (2)	0.81998 (7)	0.0232 (3)	
H9	0.4319	0.1630	0.7955	0.028*	
C10	0.62084 (13)	−0.02829 (19)	0.83767 (6)	0.0195 (3)	
C11	0.26270 (14)	−0.5038 (2)	0.89513 (7)	0.0261 (3)	
H11A	0.2123	−0.5685	0.8458	0.031*	
H11B	0.1856	−0.4250	0.9232	0.031*	
H11C	0.3099	−0.6234	0.9291	0.031*	
C12	0.94008 (13)	0.04830 (19)	0.96715 (6)	0.0205 (3)	
H12A	0.8357	0.0451	0.9858	0.025*	
H12B	0.9662	0.2060	0.9566	0.025*	

### Atomic displacement parameters (Å^2^)

**Table:**

	*U*^11^	*U*^22^	*U*^33^	*U*^12^	*U*^13^	*U*^23^
O1	0.0200 (4)	0.0209 (4)	0.0243 (4)	0.0001 (3)	0.0000 (3)	0.0041 (3)
C2	0.0187 (5)	0.0238 (6)	0.0194 (6)	−0.0003 (4)	0.0012 (4)	0.0010 (4)
N3	0.0195 (5)	0.0195 (5)	0.0180 (5)	−0.0007 (4)	0.0001 (4)	−0.0003 (4)
C4	0.0204 (6)	0.0183 (6)	0.0195 (5)	0.0008 (4)	0.0001 (4)	−0.0005 (4)
C5	0.0202 (6)	0.0207 (6)	0.0155 (5)	0.0008 (4)	0.0003 (4)	−0.0030 (4)
C6	0.0238 (6)	0.0195 (6)	0.0179 (5)	0.0005 (4)	0.0010 (4)	−0.0018 (4)
C7	0.0217 (6)	0.0251 (6)	0.0193 (5)	−0.0018 (5)	0.0028 (4)	−0.0048 (5)
C8	0.0176 (6)	0.0291 (7)	0.0263 (6)	0.0026 (5)	0.0000 (4)	−0.0029 (5)
C9	0.0234 (6)	0.0217 (6)	0.0239 (6)	0.0035 (5)	−0.0008 (4)	0.0004 (5)
C10	0.0212 (6)	0.0209 (6)	0.0164 (5)	−0.0013 (4)	0.0012 (4)	−0.0023 (4)
C11	0.0216 (6)	0.0295 (7)	0.0275 (6)	−0.0021 (5)	0.0040 (5)	−0.0023 (5)
C12	0.0205 (5)	0.0196 (6)	0.0208 (6)	0.0012 (4)	−0.0007 (4)	−0.0017 (4)

### Geometric parameters (Å, °)

**Table:**

O1—C10	1.3755 (14)	C6—H6	0.9300
O1—C2	1.4525 (13)	C7—C8	1.3958 (18)
C2—N3	1.4291 (14)	C7—C11	1.5052 (16)
C2—H2A	0.9700	C8—C9	1.3808 (17)
C2—H2B	0.9700	C8—H8	0.9300
N3—C12	1.4663 (14)	C9—C10	1.3910 (16)
N3—C4	1.4701 (14)	C9—H9	0.9300
C4—C5	1.5134 (15)	C11—H11A	0.9600
C4—H4A	0.9700	C11—H11B	0.9600
C4—H4B	0.9700	C11—H11C	0.9600
C5—C6	1.3926 (16)	C12—C12^i^	1.521 (2)
C5—C10	1.3928 (16)	C12—H12A	0.9700
C6—C7	1.3905 (16)	C12—H12B	0.9700
			
C10—O1—C2	113.49 (8)	C6—C7—C11	121.71 (11)
N3—C2—O1	113.69 (8)	C8—C7—C11	120.52 (10)
N3—C2—H2A	108.8	C9—C8—C7	121.23 (10)
O1—C2—H2A	108.8	C9—C8—H8	119.4
N3—C2—H2B	108.8	C7—C8—H8	119.4
O1—C2—H2B	108.8	C8—C9—C10	119.98 (11)
H2A—C2—H2B	107.7	C8—C9—H9	120.0
C2—N3—C12	113.12 (9)	C10—C9—H9	120.0
C2—N3—C4	108.35 (8)	O1—C10—C9	117.26 (10)
C12—N3—C4	113.04 (8)	O1—C10—C5	122.45 (10)
N3—C4—C5	111.94 (9)	C9—C10—C5	120.28 (11)
N3—C4—H4A	109.2	C7—C11—H11A	109.5
C5—C4—H4A	109.2	C7—C11—H11B	109.5
N3—C4—H4B	109.2	H11A—C11—H11B	109.5
C5—C4—H4B	109.2	C7—C11—H11C	109.5
H4A—C4—H4B	107.9	H11A—C11—H11C	109.5
C6—C5—C10	118.56 (10)	H11B—C11—H11C	109.5
C6—C5—C4	122.21 (10)	N3—C12—C12^i^	110.89 (11)
C10—C5—C4	119.22 (10)	N3—C12—H12A	109.5
C7—C6—C5	122.18 (11)	C12^i^—C12—H12A	109.5
C7—C6—H6	118.9	N3—C12—H12B	109.5
C5—C6—H6	118.9	C12^i^—C12—H12B	109.5
C6—C7—C8	117.77 (10)	H12A—C12—H12B	108.1

Symmetry codes: (i) −*x*+2, −*y*, −*z*+2.

### Hydrogen-bond geometry (Å, °)

**Table:**

*D*—H···*A*	*D*—H	H···*A*	*D*···*A*	*D*—H···*A*
C2—H2A···O1^ii^	0.97	2.57	3.425 (1)	147

Symmetry codes: (ii) −*x*+3/2, *y*−1/2, −*z*+3/2.
